# Characterizing the gene–environment interaction underlying natural morphological variation in *Neurospora crassa* conidiophores using high-throughput phenomics and transcriptomics

**DOI:** 10.1093/g3journal/jkac050

**Published:** 2022-02-28

**Authors:** Emily K Krach, Michael Skaro, Yue Wu, Jonathan Arnold

**Affiliations:** Genetics Department, University of Georgia, Athens, GA 30602, USA; Institute of Bioinformatics, University of Georgia, Athens, GA 30602, USA; Institute of Bioinformatics, University of Georgia, Athens, GA 30602, USA; Genetics Department, University of Georgia, Athens, GA 30602, USA; Institute of Bioinformatics, University of Georgia, Athens, GA 30602, USA

**Keywords:** *Neurospora crassa*, natural variation, conidiophore, vegetative development, morphology, sporulation, germination, convolutional neural network, phenomics, transcriptomics

## Abstract

*Neurospora crassa* propagates through dissemination of conidia, which develop through specialized structures called conidiophores. Recent work has identified striking variation in conidiophore morphology, using a wild population collection from Louisiana, United States of America to classify 3 distinct phenotypes: Wild-Type, Wrap, and Bulky. Little is known about the impact of these phenotypes on sporulation or germination later in the *N. crassa* life cycle, or about the genetic variation that underlies them. In this study, we show that conidiophore morphology likely affects colonization capacity of wild *N. crassa* isolates through both sporulation distance and germination on different carbon sources. We generated and crossed homokaryotic strains belonging to each phenotypic group to more robustly fit a model for and estimate heritability of the complex trait, conidiophore architecture. Our fitted model suggests at least 3 genes and 2 epistatic interactions contribute to conidiophore phenotype, which has an estimated heritability of 0.47. To uncover genes contributing to these phenotypes, we performed RNA-sequencing on mycelia and conidiophores of strains representing each of the 3 phenotypes. Our results show that the Bulky strain had a distinct transcriptional profile from that of Wild-Type and Wrap, exhibiting differential expression patterns in *clock-controlled genes* (*ccgs*), the conidiation-specific gene *con-6*, and genes implicated in metabolism and communication. Combined, these results present novel ecological impacts of and differential gene expression underlying natural conidiophore morphological variation, a complex trait that has not yet been thoroughly explored.

## Introduction

The classic filamentous fungus *Neurospora crassa* reproduces vegetatively through the development of macroconidiophores (conidiophores). These specialized structures serve to generate haploid macroconidia (conidia) that sporulate and germinate to propagate the asexual life cycle throughout an environment. Conidiophore development in *N. crassa* is well understood following decades of study in the laboratory. Briefly, the conidiation process begins with perpendicular growth of an aerial hypha. The filament undergoes minor and major constriction budding to generate spores that will eventually break off as mature conidia (Springer and Yanofsky 1989). Environmental cues such as desiccation, aeration, nutrient deprivation, and light are required for induction of this process, which is under strict circadian regulation (Sargent and Kaltenborn 1972; Nelson *et al.* 1975; Loros and Dunlap 2001). Many genes guiding conidiophore development have been identified, and their expression has been characterized throughout the roughly 12-h developmental timeline (Springer and Yanofsky 1989; Greenwald *et al.* 2010). While these environmental, temporal, and genetic signals of conidiophore development are well understood, little is known about the morphological variation of these structures, particularly in natural populations.

Conidiophore architectural variation has just recently been explored in a wild population collection of *N. crassa* isolates from Louisiana, United States of America (Krach *et al.* 2020). This work identified 3 novel and distinct phenotypes, named Wild-Type (WT), Wrap, and Bulky, where WT conidiophores formed linear chains of conidia, Wrap conidiophores wrapped around and/or stuck to hyphal filaments, and Bulky conidiophores formed tight clusters (Krach *et al.* 2020). These phenotypes were found to be upheld throughout the duration of their development and had a significant impact on resulting “spore shadows,” or distributions of spore dispersal distances. This suggests that conidiophore architectural phenotype may affect colonization capacity of the organism, a hypothesis further explored in this study.

Here, using the same wild population collection of 21 strains, we reinforce that conidiophore morphology impacts spore dispersal distances on a larger scale, also affecting the maximum distance a conidium may travel. Furthermore, we show that conidiophore phenotype influences both germination rates and germination times of conidia on growth media containing different carbon sources, particularly in the Bulky strain. Applying methods developed in Krach *et al.* (2020), we crossed homokaryotic strains for each phenotype and machine-classified conidiophores of the resulting progeny. These phenotypic counts were used to fit a model where at least 3 genes and 2 epistatic interactions contributed to conidiophore morphology, a complex trait with an estimated heritability of 0.47. To identify genes underlying these conidiophore phenotypes, we performed RNA-sequencing (RNA-seq) on both mycelia and conidiophores of a representative strain for each phenotypic group. Our results show that the Bulky strain has a unique transcriptional profile from WT and Wrap, exhibiting the most striking differential expression patterns in *clock-controlled genes* (*ccg-1*, *ccg-2*, *ccg-14*), metabolic genes responsive to starvation (*acu-6*, NCU04482), genes involved in communication (*doc-1*, *doc-2*, *plp-1*, *plp-2*), and the conidiation-specific gene, *con-6*. To our knowledge, this is the first report of genes implicated in natural morphological variation of *N. crassa* conidiophores. Together, this work lends insight to phenotypic variation of the conidiophore and its robustness to environmental perturbations, as well as genetic differences underlying this variation in natural populations.

## Materials and methods

### Strains and media

Wild Louisiana isolates were obtained from the Fungal Genetics Stock Center (FGSC, Manhattan, KS, USA) and are listed in Table 1. Strains were maintained on 1.8% glucose/1.8% fructose/1.5% agar slants with 1X Vogel’s media and recommended biotin and trace element supplements (hereon referred to as standard VM; Davis and de Serres 1970).

**Table 1.** **Strains originally collected in nature by David Perkins and colleagues and available in the FGSC were used in this study (Krach *et al.***  2020**).**

Strain number	FGSC	Perkins	Mat	Strain provenance	Collection site	Substrate/annotation
Wild Strains						
D110	8870	4448	A	Dettman, J.	Franklin, LA	Sugarcane
D111	8871	4449	a	Dettman, J.	Franklin, LA	Sugarcane
D112	8872	4453	A	Dettman, J.	Franklin, LA	Sugarcane
D114	8874	4464	A	Dettman, J.	Franklin, LA	Sugarcane
D116	8876	4481	a	Dettman, J.	Franklin, LA	Sugarcane
D118	8878	4491	a	Dettman, J.	Franklin, LA	Sugarcane
JW09	2229		A	Welch, J.	Welsh, LA	Burned grass
JW10	2229		A	Welch, J.	Welsh, LA	Burned grass
JW59	3200		a	Welch, J.	Coon, LA	Burned stumps
JW66	3211		a	Welch, J.	Sugartown, LA	Pine burn
JW70	3199		A	Welch, J.	Coon, LA	Burned stumps
JW75	3943		a	Welch, J.	Houma, LA	Sugarcane burn
	847		A	Lein	Louisiana	Sugarcane burn
D113	8873	4454	a	Dettman, J.	Franklin, LA	Sugarcane
D119	8879	4500	a	Dettman, J.	Franklin, LA	Sugarcane
JW20	3212		A	Welch, J	Ravenswood, LA	Bonfire
JW76	3943		a	Welch, J	Houma, LA	Sugarcane burn
JW159	2221		a	Welch, J	Houma, LA	Sugarcane burn
JW160	2222		A	Welch, J	Iowa, LA	Grass burn
JW162	2223		a	Welch, J	Iowa, LA	Grass burn
JW164	2224		a	Welch, J	Marrero, LA	Wood burn
JW167	2228		a	Welch, J	Roanoke, LA	Grass burn
OR74A	2489		A	FGSC	Marrero, LA	Unknown

One of these strains (OR74A) is the genome reference strain (Galagan *et al.* 2003).

### Sporulation experiment

Representative strains for each phenotypic class (FGSC8872, FGSC8876, and FGSC3943) were inoculated on standard VM and incubated at 30°C for 30 h. Mycelia were then harvested onto a 60-mm diameter nitrocellulose membrane with 0.45-μm pore size (Whatman Protran BA-85, Maidstone, England), inverted onto a new plate with media as described above, and set under the light for 20 h before being transferred to a new environment for sporulation.

To explore sporulation on a larger scale, this “new environment” was 18 × 18 inch plastic cake platters repurposed as Petri dishes (Fineline Settings, Middletown, NY, USA). The platters and their clear plastic lids were sterilized with ethanol and UV light before use. The nitrocellulose membranes containing isolated conidiophores were placed at the center of each cake platter, where sorbose + fructose + glucose (SFG) media (Davis and de Serres 1970) surrounded a 60-mm diameter blank space now occupied by the membrane. Each platter was covered with a lid through which light could penetrate and placed under the light for 4 days to allow sporulation, germination, and subsequent colonial growth. Pictures of each plate were taken with an iPhone XS, and ImageJ (Schneider *et al.* 2012) was used to measure the distance from the center of each nitrocellulose membrane to the center of a colony, sampling up to 75 colonies per platter. Three biological replicates of each strain were performed.

### Germination assay

Representative strains used for each phenotypic group were FGSC8872 for WT, FGSC8876 for Wrap, and FGSC2229 for Bulky. Cultures were grown up on standard VM for 5 days under light (Davis and de Serres 1970). Conidia were then suspended in water, counted using a Cellometer Auto 2000 (Nexcelom, Inc. Lawrence, MA USA), and diluted to roughly 1.00 × 10^3^ cells/ml (Case *et al.* 2014). One hundred microliters of conidial suspensions were pipetted onto 1.5% agar plates with 1% sorbose, 1X Vogels medium, recommended biotin and trace element supplements, and various carbon sources as follows: fructose and glucose (0.1% and 0.01%), mannose (1%, 0.1%, 0.01%), and xylose (1%, 0.1%, 0.01%). Plates were kept at 30°C for up to 7 days and checked daily for conidial germination, as germination time often varied with strain and medium. Colonies were counted once conidia germinated. Each strain was plated in triplicate for each medium condition, always alongside a positive control plate with standard VM.

We counted the number of colonies on each plate and calculated the average among 3 replicates. To calculate germination rates, we divided the average number of colonies per strain and condition by the expected colony count (based on conidial suspension concentration) × 100%.

To image conidiophores on 0.1% Mannose and 0.1% Xylose media (recipes described above), we left plates at 30°C for an additional 3 days after counting colonies to allow sufficient conidiation. Brightfield images were taken at 20× using the microscope and augmentation methods described below (Microscopy and Image Deconvolution).

### Crosses and progeny screening

Crosses were performed in the dark on cornmeal crossing medium (Perkins 2006), after which ascospores were plated on SFG medium. Colonies were subsequently picked to isolate random ascospore progeny. Wild strains selected for initial crossing are listed in Table 2. Crosses were conducted in duplicate with 25 progeny selected from each cross.

**Table 2.** Wild isolates selected for first set of crossing to generate F1s.

Parent (A)	Phenotype		Parent (a)	Phenotype		Offspring
FGSC8872	WT 77.78%	X	FGSC3943	Bulky 48.78%	→	#42, 43
FGSC8872	WT 77.78%	x	FGSC8876	Wrap 53.85%	→	#151, 156
FGSC2229	Bulky 46.55%	x	FGSC8876	Wrap 53.85%	→	#83, 91

Some of the Wild isolates in Table 1 were crossed to form an F1 as described in *Materials and Methods* to make them homokaryotic.

Because the original wild strains may possibly be heterokaryotic, we crossed representative strains from each phenotypic group (FGSC8872 for WT, FGSC8876 for Wrap, FGSC2229 and FGSC3943 for Bulky) to generate homokaryotic F1s. We then crossed F1s that represented each phenotypic group with the highest penetrance, or fraction of conidiophores displaying a particular phenotype. F1s selected for crossing are listed in Table 3. F2 ascospores were plated on SFG medium as described above and picked to isolate 30 random ascospore progeny from each cross.

**Table 3.** F1s selected for crossing to generate F2s.

Parent (A)	Penetrance^*a*^		Parent (a)	Penetrance
WT #151	100%	×	Bulky #91	100%
WT #151	100%	×	Wrap #43	80%
Wrap #156	83%	×	Bulky #91	100%

The progeny of these crosses (as described in the *Materials and Methods*) are shown in Fig. 5.

*^a^* Fraction of offspring displaying a particular phenotype. The phenotype is defined in the first or fourth column.

To isolate and image conidiophores in a high-throughput manner while preventing fusion of different progeny sharing a plate, each F2 strain was inoculated on a 1 ml standard VM agar droplet (Davis and de Serres 1970). Each 150 × 15 mm Petri dish contained 8 agar droplets evenly spaced roughly 2.5 cm apart. Each droplet was inoculated with progeny conidia and incubated at 30°C for 20 h to allow sufficient mycelial growth without hyphal fusion between droplets. Each droplet was then harvested onto a separate nitrocellulose membrane with 0.45-µm pore size (Whatman Protran BA-85, Maidstone, England). Each membrane was inverted onto a new agar droplet as described above and placed under light for aerial hyphae to penetrate the membrane. After 25 h, membranes were removed from the agar and secured on a flat surface for imaging of conidiophores (Bailey-Shrode and Ebbole 2004; Krach *et al.* 2020).

### Microscopy and image deconvolution

Nitrocellulose membranes containing conidiophores were visualized on an inverted microscope (Axio Observer A1, Carl Zeiss Microscopy, LLC, Thornwood, NY, USA) at 20× magnification and brightfield images were taken with a charge-coupled device camera (AxioCam HRm, Carl Zeiss Microscopy, LLC, Thornwood, NY, USA). Multiple z-slices were captured and overlaid in ImageJ (Schneider *et al.* 2012) to convey a complete representation of the 3D conidiophore structure. Augmentation including contrast enhancement and noise and background subtraction was conducted on image stacks to isolate conidiophores from underlying mycelia and/or aerial hyphae.

Classification of conidiophores was carried out based on the pretrained convolutional neural network (Krach *et al.* 2020). Starting from the classifier published in Krach *et al.* (2020), we used 327 additional images to fine-tune and adapt the published model for the new F2 dataset. The 3 phenotypes were equally distributed among the image samples and train-validation-test separation was approximately 8:1:1. The model was trained with an additional 100 epochs, and the best model was selected by the best accuracy in the validation set. Other hyperparameters and model evaluations were kept the same as in the original publication (Krach *et al.* 2020).

### RNA extraction

Previous work has shown that growing fungi on solid medium overlayed with a nylon membrane facilitates harvest of the mycelium, produces sufficient biomass, and enhances RNA quality (Schumann *et al.* 2013). Large Petri dishes (150 × 15 mm) with standard VM were covered with a Hybond XL Nylon membrane (Amersham, Buckinghamshire, UK). Membranes were inoculated with conidia from representative wild strains (FGSC8872, FGSC8876, and FGSC2229) and placed at 30°C for 30 h to allow for mycelial growth. Mycelia were then harvested with a sterile razor blade into a microcentrifuge tube and immediately frozen with liquid nitrogen for later RNA extraction. Four biological replicates of each strain were grown and harvested.

Conidiophore cultures were first grown following the protocol above. At the 30-h time point, mycelia were covered with a 0.45-μm pore size nitrocellulose membrane (Whatman Protran BA-85, Maidstone, England) and placed under the light for 20 h. Aerial hyphae penetrated the nitrocellulose membrane, allowing conidiophore isolation from this top layer. Conidiophores were then harvested with a sterile razor blade and immediately frozen with liquid nitrogen for RNA extraction. Four biological replicates of each strain were grown and harvested. The method was adapted from Krach *et al.* (2020) and Schumann *et al.* (2013).

Mycelia and conidiophore samples were later ground to fine powder using the Cellcrusher tissue pulverizer (Cellcrusher, Cork, Ireland) submerged in liquid nitrogen. Samples were transferred to a new microcentrifuge tube and kept frozen. Total RNA was later isolated and suspended in RNase-free water using the Qiagen RNeasy Plant Mini Kit (QIAGEN, Inc., Valencia, CA, USA) following the protocol outlined by the manufacturer. RNA integrity was assessed using the Agilent Bioanalyzer and RNA concentration was quantified using the fluorometric Qubit analyzer at the Georgia Genomics and Bioinformatics Core.

### RNA library preparation

Libraries were prepared according to the KAPA Stranded RNA-seq Kit. The libraries were then pooled and sequenced on a NextSeq2000 instrument to generate paired-end reads. Library preparation, pooling, and sequencing were conducted at the Georgia Genomics and Bioinformatics Core.

### RNA-seq data analysis

Sequencing reads were demultiplexed by BaseSpace (Illumina). Reads were trimmed from the adaptor sequences using the cutadapt software (Martin 2011) and aligned to the *N.* *crassa* genome (NC12) following star quantification mRNA-seq pipeline. Gene expression data were analyzed in R version (4.0.3). The differentially expressed genes were measured using Bioconductor: DeSeq2 (Love *et al.* 2014). Differential gene expression was measured between cell type and strain. Power estimation for differential expression analysis was conducted using the Vanderbilt power calculation for RNA-seq experiment Shiny app (Guo *et al.* 2014). Genes were filtered for an adjusted *P*-value and absolute fold change of 10e^−5^ and absolute log_2_FC > 3.0, respectively.

Differentially expressed genes were extracted from the results table generated in the DESeq2 pipeline. Expression values for the top 50 most significantly differentially expressed genes were used to hierarchically cluster samples. The DESeq2 package was used to conduct principal component analysis (PCA) and generate PCA plots. Hierarchical clustering was used to discriminate sample phylogeny in the experimental setting. Pretty Heatmaps package (Kolde 2018) was used to visualize the expression patterns of the most significantly differentially expressed genes for each experimental design.

## Results

### Conidiophore architectural phenotype may impact colonization capacity of *N. crassa* by affecting the maximum dispersal distance of released conidia

Previous work showed that conidiophore architectural phenotype may play a role in colonization capacity of the organism by affecting spore dispersal distance (Krach *et al.* 2020). Dispersal experiments to characterize population structure have been extensively used in population genetics (Dobzhansky and Wright 1943; Trapnell and Hamrick 2005; Hamrick and Trapnell 2011). For example, neighborhood size is directly related to the dispersal distribution (Dobzhansky and Wright, 1943; Wright, 1968). A variety of studies have been recently carried out on spore dispersal (Roper et al. 2008, 2010; Fritz *et al.* 2013). A key element to the design of these experiments is the size of the grid on which dispersal is measured (Powell and Dobzhansky 1976). We wanted to scale up our previous spore shadow experiment to explore spore dispersal over a larger area (Krach *et al.* 2020). Limiting the boundaries of a dispersal experiment to measure neighborhood size introduces biases in specifying the distribution of dispersal distances of the organism (Lemke 1985). To expand these boundaries, we allowed conidiophores of each architectural phenotype to sporulate and germinate on 0.1% SFG medium, reinventing an 18 × 18 inch plastic cake platter as a large Petri dish.

**Fig. 1.**
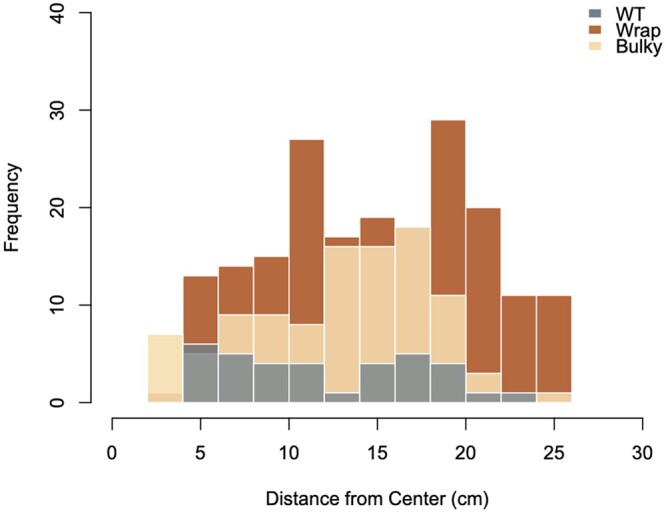
Distribution of spore dispersal distances scaled up. Histogram of distances in centimeters to the center of each colony from the center of each nitrocellulose membrane. Total colony counts combining 3 replicates are as follows: WT = 35, Wrap = 195, and Bulky = 104. Strains selected for each phenotypic group were as follows: FGSC8872 for WT, FGSC8876 for Wrap, and FGSC3943 for Bulky. Results of 2-sample Kolmogorov–Smirnov tests for each phenotype pair are as follows: WT-Wrap D = 0.24982, *P* = 0.04926; Wrap-Bulky D = 0.2109, *P* = 0.004794; WT-Bulky D = 0.18709, *P* = 0.2734.

Consistent with the findings in Krach *et al.* (2020), fewer colonies (*n* = 35) developed on the cake platter (see *Materials and* *Methods*) from WT conidiophores at the center of the cake platter compared to that of Wrap and Bulky (*n* = 195 and *n* = 104, respectively; see Supplementary Fig. 1 and histograms in Fig. 1). The distribution of spore dispersal distances by the Wrap conidiophores was significantly different from that of both WT (*P = *0.04926) and Bulky (*P = *0.004794) phenotypes, found by 2-sample Kolmogorov–Smirnov tests (Fig. 1; Kendall and Stuart 1979). No significant difference was found in spore dispersal distance distributions between the WT and Bulky groups (*P = *0.2734). Both findings are consistent with sporulation patterns observed at a smaller scale, indicating that the spore shadows displayed by each conidiophore phenotype are upheld in this new, larger environment (Krach *et al.* 2020). Taking advantage of this larger controlled environment, we then sought to investigate whether the conidiophore architectural phenotype had an impact on the maximum distance a conidium could travel and subsequently germinate.

Previous work showed that WT spores traveled the least distance following dispersal (Krach *et al.* 2020). The null hypothesis examined is whether the maximum distance traveled by Wrap and Bulky could be considered to be drawn from the same distribution of the maximum distance squared traveled by WT (Table 4). A Shapiro–Wilk test of normality (Royston 1995) was performed on WT (SW = 0.9313, *P *= 0.0306)—departures from normality are not strong. To a first approximation, it is reasonable to suppose that spore dispersal coordinate is normally distributed (Powell and Dobzhansky 1976). The distribution of standardized distance squared is then chi-squared with 1 degree of freedom. The largest distance traveled has a known distribution, and the probability that the largest distances seen in Wrap and Bulky are drawn from the dispersal distribution of WT is extremely unlikely (David 1981).

**Table 4.** Maximum spore dispersal distances traveled by Wrap and Bulky are significantly different from WT.

Strain (phenotype)	Maximum distance	Tail probability of the WT distribution of the largest rank of distance squared
FGSC8872 (WT)	23.50 cm	—
FGSC8876 (Wrap)	25.78 cm	3.80 × 10^−64^
FGSC3943 (Bulky)	24.16 cm	2.15 × 10^−51^

Maximum distance in centimeters among 3 biological replicates traveled by a germinating conidium of each phenotype. For all 3 values, the mean movement ($\overset{-}{X}$ = 4.729) was subtracted, the sample variance (*S*^2^ = 2.165) was used to standardize, and the resulting z-value (z = (X$\overset{-}{X}$)/s), squared. The resulting *z*^2^ is then approximately chi-squared in distribution with 1 degree of freedom. The tail probability for the distribution of the largest rank from this chi-squared distribution was then computed as if these *z*^2^ values were all order statistics from the same distribution (David 1981).

### Spores from conidiophores with different architectural phenotypes show distinct germination patterns on different carbon sources

**Fig. 2.**
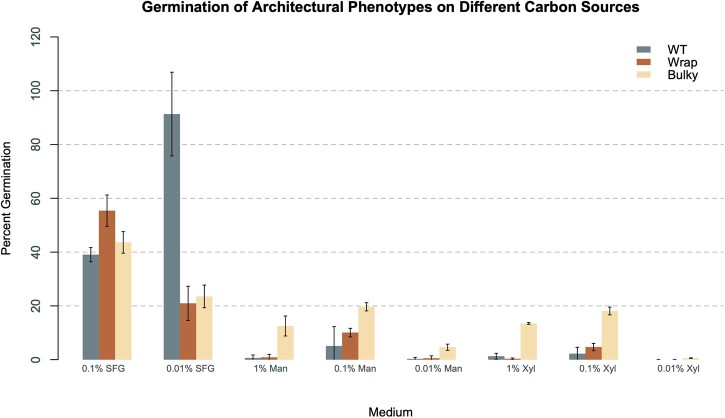
Germination rates of architectural phenotypes on different carbon sources. Germination rates of conidia from each phenotypic group (FGSC8872 for WT, FGSC8876 for Wrap, and FGSC2229 for Bulky) on media containing varying concentrations of different carbon sources.

We sought to further explore the potential impact of conidiophore phenotype on environmental colonization through the lense of germination. To do this, we plated known amounts of conidia from a WT, Wrap, and Bulky strain (FGSC8872, FGSC8876, and FGSC2229, respectively) onto media containing 1% sorbose, a monosaccharide known to limit the organism to colonial growth. Each colony on a plate represents germination of 1 conidium. We varied the media to assess the germination behavior of each conidiophore phenotype on different carbon sources: Fructose/Glucose, Mannose, and Xylose. In addition to the different carbon sources, we also explored varying concentrations of each carbon source. Germination rates were determined from colonies counted/expected colonies × 100% and are depicted below in Fig. 2.

First, we compared spore germination rates on different carbon sources. When compared to different concentrations of the same carbon source, all strains show the highest germination rate with a sugar concentration of 0.1%, with the one exception of WT on 0.01% SFG (Fig. 2). Interestingly, spores from WT conidiophores show a jump in germination from 39% to 91% when fructose and glucose are decreased by an order of magnitude, whereas Wrap and Bulky groups both show lower germination rates. This pattern exhibited by WT indicates a starvation response that is unique to this phenotypic group on SFG medium. In all concentrations of media containing mannose and xylose, Bulky spores consistently show a significantly elevated germination rate compared to the other 2 groups. This is a pattern unique to these 2 carbon sources and is not observed at either concentration of SFG medium.

**Fig. 3.**
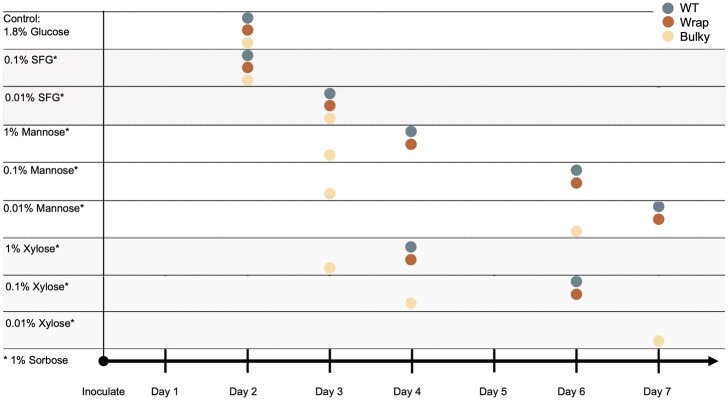
Germination timeline of architectural phenotypes on different carbon sources. Germination times of conidia from each conidiophore phenotype (FGSC8872 for WT, FGSC8876 for Wrap, and FGSC2229 for Bulky) on different media conditions at 30°C.

In addition to germination rate, germination time also varies with conidiophore phenotype under some medium conditions (Fig. 3). All 3 phenotypic groups germinated synchronously on SFG medium, as well as on the positive control of 1.8% Glucose Vogel’s Medium. However, on all concentrations of mannose and xylose, Bulky conidia germinated earlier than the other 2 phenotypic groups, which remained synchronous. Interestingly, this unique temporal behavior exhibited by Bulky conidia is consistent with the uniqueness of higher germination rates observed by Bulky on these same carbon sources.

On all carbon sources, germination of each strain is delayed as concentration of the sugar decreases. This is true in all cases, except for Bulky on 0.1% and 1% Mannose where germination occurs on Day 3 under both conditions. The germination timeline is very predictable on mannose and xylose (Fig. 3). The key points are that Bulky is behaving differently from WT and Wrap with regard to germination rate and timeline on some media (mannose and xylose), and WT is behaving differently on other media (SFG; Figs. 2 and 3).

**Fig. 4.**
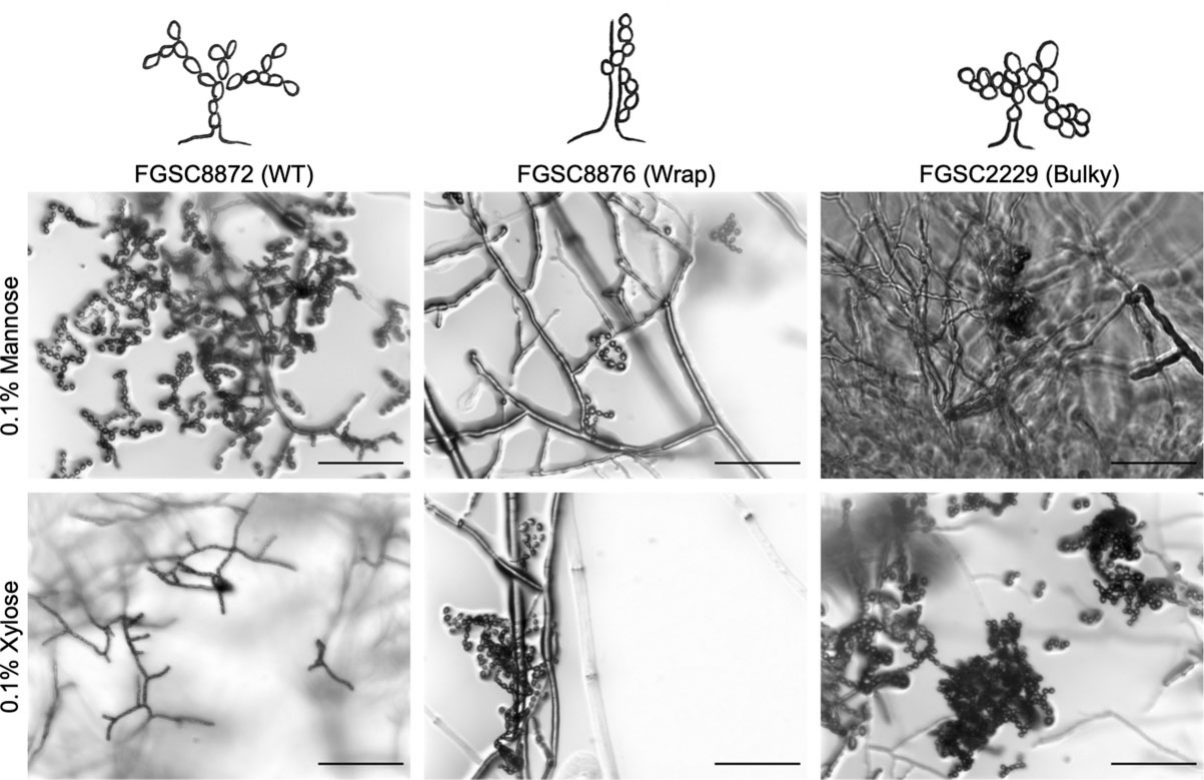
Conidiophore phenotypes on media containing mannose and xylose. Conidiophore architectural phenotypes, depicted in drawings above each column label, are upheld on media containing 0.1% mannose (top row) and 0.1% xylose (bottom row) as a carbon source. Representative strains used were FGSC8872 for WT, FGSC8876 for Wrap, and FGSC2229 for Bulky. Scale bar, 100 µm.

After characterizing germination on these new carbon sources, we were curious if conidiophore architectural phenotype was upheld once conidiation occurred. We imaged conidiophores of each strain on 0.1% mannose and 0.1% xylose, as germination rates were highest with this sugar concentration for all 3 strains. On both 0.1% mannose and 0.1% xylose, WT, Wrap, and Bulky architectural phenotypes were upheld in the conidiophores (Fig. 4).

### Crosses between homokaryotic F1s suggest at least 3 genes contribute to conidiophore architectural phenotype

Previous work has shown that at least 2 genes contribute to the conidiophore architectural phenotype, and the trait has an estimated heritability of 0.23 (Krach *et al.* 2020). This model was based on progeny phenotype counts from crosses between original Louisiana wild isolates. Because wild strains may possibly be heterokaryotic, this inheritance model would be more robust by crossing homokaryotic F1s, quantifying conidiophore phenotypes of the resulting F2, and fitting the model to those phenotypic ratios.

**Fig. 5.**
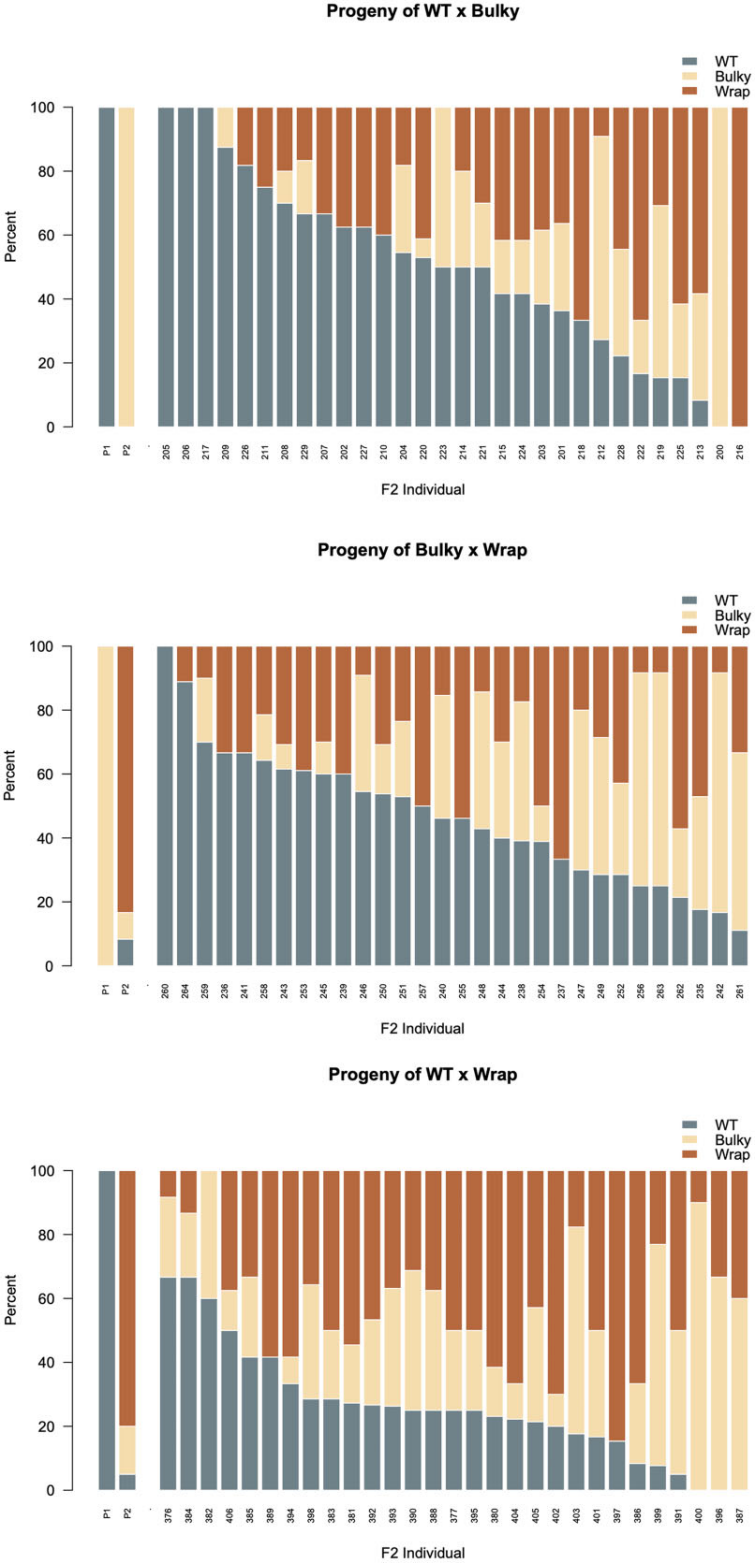
Percent of conidiophore phenotypes observed in F1 parents and resulting F2 progeny. Crosses were conducted between majority WT × Bulky (top), Bulky × Wrap (middle), and WT × Wrap (bottom) F1s, whose phenotypic ratios are depicted as the 2 left most bars on each plot. Phenotype ratios of the resulting F2 progeny are plotted as the following 30 bars.

We selected F1 parents with the greatest penetrance, or fraction of conidiophores displaying a particular conidiophore phenotype, and conducted crosses between groups. We then selected 30 random ascospore progeny from each cross. Conidiophores from these F2 were isolated, imaged, and classified using the automated classification method developed and successfully applied in Krach *et al.* (2020). To accommodate the changes of different batches and generations, we fine-tuned the classification model with 327 images and evaluated the classification performance (Supplementary Table 1). The phenotypic ratios of each F1 parent and the resulting F2 progeny are depicted below in Fig. 5 and summed in Table 5.

**Table 5.** F2 phenotype counts.

	A (WT)	B (Wrap)	C (Bulky)
A × B	$N_{11}=103$	$N_{12}$ =167	$N_{13}=126$
B × C	$N_{21}=142$	$N_{22}$ = 86	$N_{23}$ = 58
A × C	$N_{31}=170$	$N_{32}$ =108	$N_{33}$ =88

Multinomial counts of progeny phenotypes from each of the 3 crosses in Table 3. The phenotypic counts were generated by classifying the offspring by conidiophore phenotyping by machine learning (see *Materials and Methods*).

As seen in Fig. 5, there are fewer WT phenotypes in the cross of WT × Wrap than in the other 2 crosses as if Wrap is “standing upon” WT. Only 26% of the WT × Wrap offspring are WT as opposed to 50% or 46% WT in the crosses of Wrap × Bulky or WT × Bulky, respectively (Table 5). Conidiophore phenotype counts (Table 5) were used to estimate inheritance models successfully fitted in Krach *et al.* (2020). The models were fitted by the Method of Maximum Likelihood (ML) using iteratively reweighted least squares (IRLS; Kendall and Stuart 1979; Asmussen *et al.* 1987). The models incorporate 3 hypothetical genes A, B, and C that may contribute to conidiophore phenotype, and each gene has an allelic effect α, β, and γ, to give rise to the WT, Wrap, and Bulky phenotypes, respectively. A cross between parents of different phenotypes includes 2 dominant genes that interact epistatically to determine the conidiophore phenotype, where interactions are depicted as αβ, βγ, and αγ. The results for ML fitting the models computed with IRLS to a relative error <10^−8^ after 9 iterations are summarized in Table 6.

**Table 6.** Fitting of hierarchy of inheritance models.

Model	χ^2^	df	*P*	χ^2^_H0_ − χ^2^_HA_	df	*P* for HA vs. H0	Notes
Full epistatic	0.00	0	—	—	—	—	—
αβ=0	13.37	1	<0.0001	13.37–0.00 = 13.37	1	<0.0001	HA = full epistatic
αβ=α=0	16.27	2	<0.0001	16.27–13.37 = 9.12	1	0.002	HA = αβ=0
βγ=0	43.42	1	<0.0001	43.42–0.00 = 43.42	1	<0.0001	HA = full epistatic
βγ=β=0	43.21	2	<0.0001	43.21–43.42	1	0.64	HA = βγ=0
αβ=β=0	29.88	2	<0.0001	29.88–13.37 = 16.51	1	<0.0001	H0 = αβ=0
αγ=0	11.11	1	0.0009	11.11–0.00 = 11.11	1	0.0009	HA = full epistatic
αγ=α=0	126.37	`	<0.0001	126.37–11.11 = 115.26	1	<0.0001	HA = αγ=0
αβ=βγ=αγ=0 additive	44.61	3	<0.0001	44.61–13.37 = 31.24	3	<0.0001	H = αβ=0
Environmental	84.27	6	<0.0001	84.27–44.61 = 39.66	6	<0.0001	HA = additive
Heritability^*a*^							H^2^ = (84.27–44.61)/84.27 = 0.47 H0 = environmental model H1 = full additive model

A nested hierarchy of inheritance models was successfully fitted with at least 3 genes controlling the conidiophore architectural phenotype to the counts of progeny phenotypes from 3 crosses (Table 5), in which the number of offspring from each cross is fixed. Nine iterations were necessary to achieve the desired error tolerance of 10^−8^ with IRLS. Recommended model was bolded along with its goodness of fit to the counts of phenotypes in crosses. A null hypothesis (H0) is tested against an alternative (HA) using the chi-squared test statistics (χ^2^) for goodness of fit with degrees of freedom (df). The models are arranged in complexity from the simplest model with no genetic effects to the most complex, the full epistatic model. In each case, a simpler model (H0) is compared to a more complex alternative model (HA) as the hierarchy is climbed in Supplementary Fig. 2. Goodness of fit was assessed by $X^{2}=\sum_{i=1}^{3}\sum_{j=1}^{3}{\left(N_{ij}-E_{ij}\right)}^{2}/E_{ij}$, and the difference of such *X*^2^s, in comparing fit of 2 models.

*^a^* Ratio of additive variation to total variation.

**Table 7.** ML estimates of allelic effects and epistatic effects in a 3-locus model of inheritance.

Parameters	αγ=0 3 Genes
α	0.14 ± 0.0130
β	0.38 ± 0.0182
γ	−0.28 ± 0.0085
αβ	−0.24 ± 0.0175
βγ	−0.55 ± 0.0175
αγ	0.00

The full epistatic model with 3 genes has 3 allelic effects and 2 epistatic interactions. The standard errors were obtained from the square roots of the diagonal elements of the inverse of the information matrix N$X^{\prime }AX$.

The models fitted in Table 6 represent a hierarchy of hypotheses about the inheritance of conidiophore phenotype varying in complexity from a model with no genetic effects at all to a full epistatic model, in which all 3 loci interact pairwise (Supplementary Fig. 2). The full epistatic model with 3 genes fitted the phenotypic ratios, and the weakest epistatic interaction was between the A (WT) and C (Bulky) genes (Table 7). There is a significant interaction αβ between gene A (WT) and gene B (Wrap) that captures the drop in phenotypic count of WT seen in Fig. 5 for the cross A(WT) × B(Wrap). We concluded that at least 3 genes contribute to conidiophore phenotype because none of the allelic effects α,β, or γ, could be dropped in the hierarchical testing (Supplementary Fig. 2). Using an additive model and the model without gene effects, we were able to estimate a heritability as the ratio of additive variation to total variation (*H*^2^) of 0.47 for this complex trait. This is an increase from our previously estimated *H*^2^ of 0.23, suggesting that using homokaryotic F2s reduced noise in the data (Krach *et al.* 2020).

### Bulky mycelia and conidiophore samples display different transcriptional profiles from that of the WT and Wrap strain

**Fig. 6.**
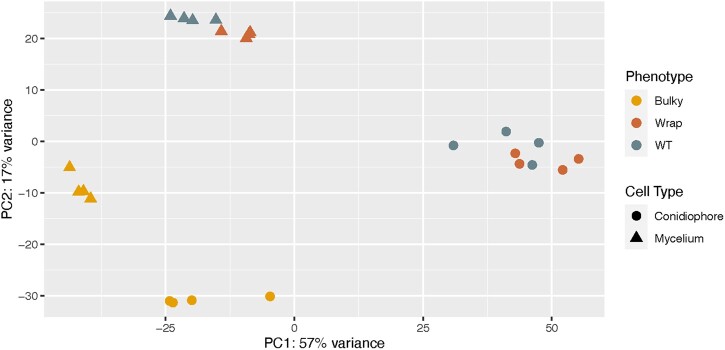
PCA of all samples characterized by RNA-seq. A 2D PCA plot was generated using normalized and variance stabilized transcript expression data (vst transformation, DESeq2) for the top 500 most variant transcripts in the dataset (measured by row variance). The percent of variance explained by each principal component is displayed on each axis. In total, 2229 samples (Hexcode: E69F00), 8872 samples (Hexcode: 68838B), and 8876 (Hexcode CD6839) cluster into 2 distinct groups on PC1 where conidiophores of 8872 and 8876 form a clear cluster away from 2229. Within the mycelium clusters, samples of 8872 and 8876 cluster together on PC2. Seventy-four percent of the total variance can be distinguished by cell type. Samples are assigned a color by strain and shape by cell type. Representative strains used were FGSC8872 for WT, FGSC8876 for Wrap, and FGSC2229 for Bulky. Four biological replicates were measured per condition.

Our inheritance model estimated at least 3 genes are involved in conidiophore architectural phenotype. To explore loci that may contribute to this complex trait, we performed RNA-seq on both mycelia and conidiophores from strains representing each conidiophore architectural phenotype (FGSC8872 for WT, FGSC8876 for Wrap, and FGSC2229 for Bulky). Clustering by PCA showed a clear grouping by cell type, as expected by previous work identifying genes differentially expressed in *N. crassa* vegetative cell types (Sachs and Yanofsky 1991; Nelson *et al.* 1997; Greenwald *et al.* 2010). Interestingly, the clustering suggests striking similarity between expression patterns of WT and Wrap mycelia, and WT and Wrap conidiophores, while Bulky cell types cluster to groups of their own (Fig. 6).

**Fig. 7.**
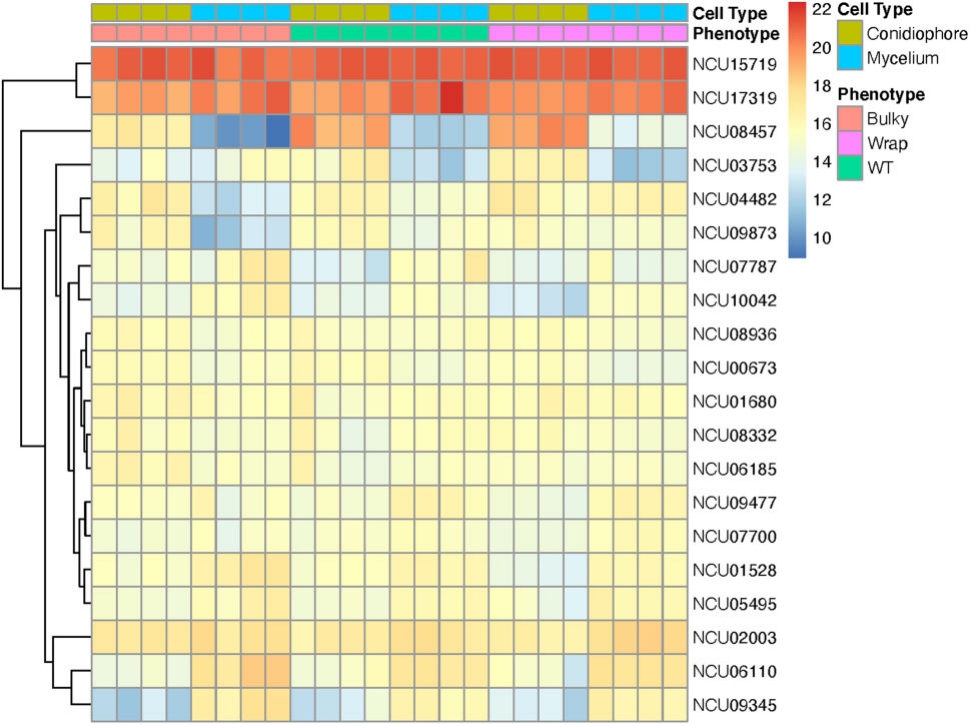
Heatmap of normalized counts. Reads were normalized in DESeq2 by the median of ratios method (Love *et al.* 2014) and are depicted for the 20 most differentially expressed genes for all strains and cell types. Each column represents a biological sample and each row a gene. Representative strains used were FGSC8872 for WT, FGSC8876 for Wrap, and FGSC2229 for Bulky. There are 4 biological replicates per condition. Differential expression model was built using a 2-factor approach estimating the phenotypes effect on cell type in the design formula. The FGSC2229 for Bulky was used as the reference in the design matrix.

Expression patterns for the 20 most differentially expressed genes are presented in Fig. 7. Of these 20 loci, 5 are *ccgs*: NCU08457 (*ccg-2*), NCU03753 (*ccg-1*), NCU07787 (*ccg-14*), NCU08936 (*ccg-15*), and NCU05495 (*ccg-16*). Interestingly, Bulky mycelia have lower *ccg-2* expression compared to WT and Wrap mycelia, just as Bulky conidiophores have lower *ccg-2* expression than both WT and Wrap conidiophores. *ccg-2* is allelic with easily wettable (*eas*) and encodes a hydrophobin critical for maintaining cell wall hydrophobicity in the conidium (Bell-Pedersen *et al.* 1992). The Bulky samples also show a unique expression pattern of *ccg-1* (Lindgren 1994), where transcription is higher in the mycelium than in the conidiophore. This contrasts with both WT and Wrap, where *ccg-1* expression is higher in the conidiophore than in the mycelium. Expression of *ccg-14* is higher in both Bulky mycelia and conidiophores when compared to the corresponding cell types of WT and Wrap samples.

Another interesting expression pattern is observed with NCU09873 (*acu-6*) and NCU04482 (uncharacterized). Both loci show strikingly lower expression in Bulky mycelia compared to all other strains and cell types. *acu-6* encodes the structural gene for phosphoenolpyruvate carboxykinase (PEPCK), and NCU04482 encodes a hypothetical protein that plays a role in amino acid metabolism (Flavell and Fincham 1968; Schmoll *et al.* 2012).

**Fig. 8.**
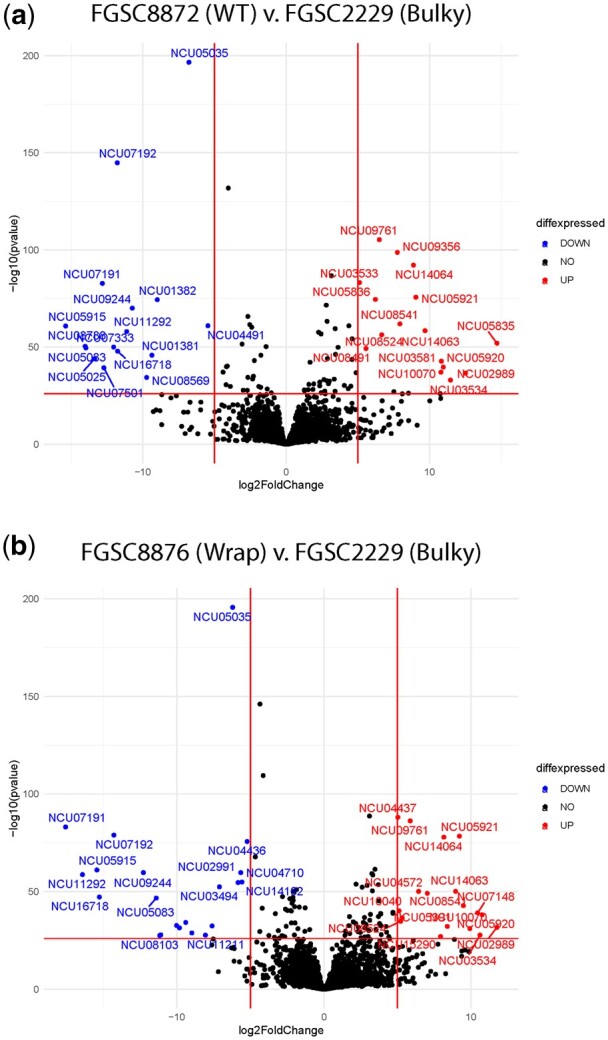
Volcano plots of significantly differently expressed genes between strains. Genes differentially expressed in WT vs Bulky strains are plotted in the top (a), and genes from the Wrap vs Bulky comparison are on the bottom (b). The log2 fold change signifies normalized expression of a gene, each represented by a dot. Each gene is color coded according to its significance, where blue genes are significantly downregulated in WT/Wrap, red genes are significantly upregulated in WT/Wrap, and black genes are not significant. Representative strains used were FGSC8872 for WT, FGSC8876 for Wrap, and FGSC2229 for Bulky.

To further explore the genes driving separation of the Bulky strain by PCA (Fig. 6), we examined genes differentially expressed when comparing WT to Bulky and Wrap to Bulky (Fig. 8). There were genes significantly up- and downregulated in both strain comparisons. Of these significantly differentially expressed genes, only 5 loci were shared by both strain comparisons, all of which were more highly expressed in Bulky than WT or Wrap. These shared genes are NCU07191 (*doc-1*), NCU07192 (*doc-2*), NCU09244 (*plp-1*), NCU09245 (*plp-2*), and NCU05035 (*vad-12*).

**Fig. 9.**
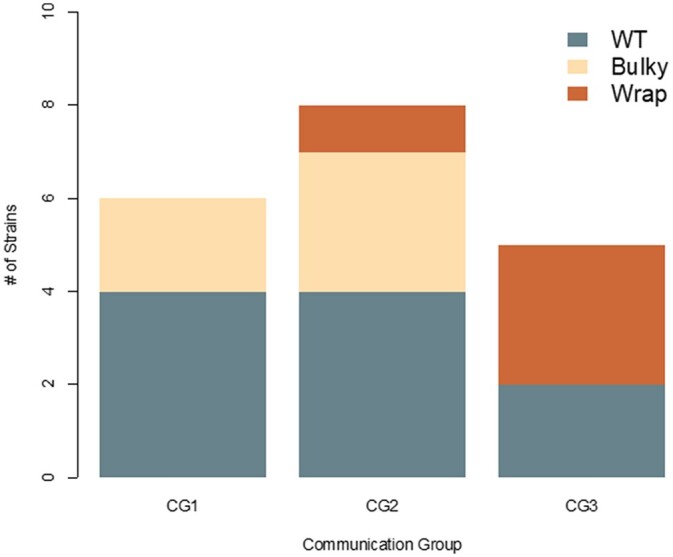
CGs of wild isolates and their conidiophore architectural phenotype. Strains are color coded by their conidiophore phenotype. There is no significant relationship between CG assignment and conidiophore phenotype determined by Chi-square test of independence (χ^2^ = 7.3388, df = 4, *P* *= *0.119).

The *doc* (*determinant of communication*) genes mediate long-distance kind-recognition, where filaments belonging to the same communication group (CG) are more likely to interact (Heller *et al.* 2016). Increased expression of *doc-1* and *doc-2* in the Bulky strain sparked the hypothesis that perhaps there was a relationship between CG and conidiophore phenotype. Previous work has characterized the CGs of 110 wild *N. crassa* isolates, including the Louisiana collection employed in this work (Heller *et al.* 2016). While the representative strains selected for RNA-seq do belong to different CGs, this pattern is not upheld when examining the complete population collection (Fig. 9). The *plp* (*palatin-like phospholipase*) genes also contribute to *N. crassa* self-recognition, triggering germling-regulated death following heterokaryon incompatibility (Heller *et al.* 2018). The final gene more expressed in Bulky than in both WT and Wrap is *vad-12*, a locus involved in vegetative asexual development that is not well characterized (Carrillo *et al.* 2017).

**Fig. 10.**
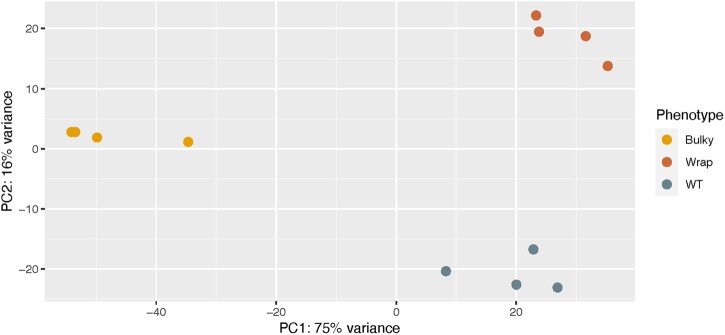
PCA of conidiophore samples characterized by RNA-seq. PCA explains 91% of the variance between the 3 strains representing conidiophore phenotypes. Samples are assigned a color according to strain. Representative strains used were FGSC8872 for WT, FGSC8876 for Wrap, and FGSC2229 for Bulky. Four biological replicates were measured per condition. Differential expression model was built using a single factor approach analyzing the phenotype in the conidiophore cell type. The FGSC2229 for Bulky was used as the reference in the design matrix.

To more specifically examine genes contributing to these morphological phenotypes, we compared expression patterns between strains for the conidiophore samples alone. PCA showed clear clustering between conidiophore samples from each of the 3 strains (Fig. 10).

**Fig. 11.**
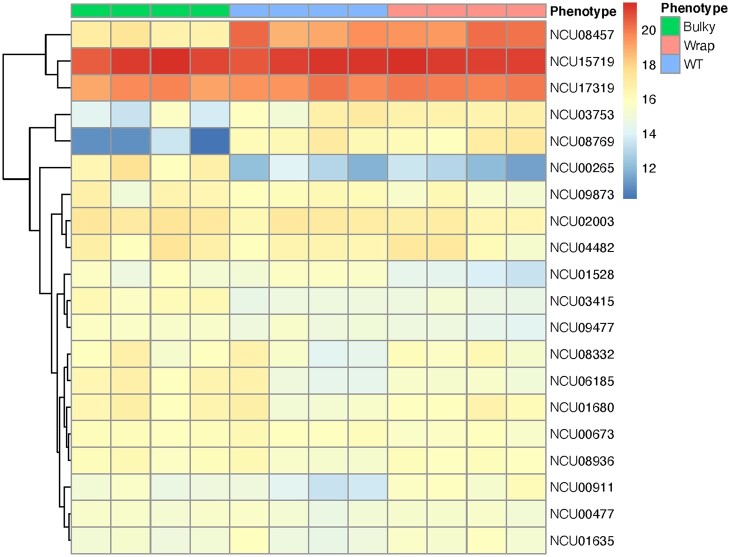
Heatmap of normalized counts in conidiophores. Read counts were normalized in DESeq2 using the median of ratios method (Love *et al.* 2014) and are depicted for the 20 most differentially expressed genes for conidiophores of all strains. Each column represents a biological sample and each row a gene. Representative strains used were FGSC8872 for WT, FGSC8876 for Wrap, and FGSC2229 for Bulky. There are 4 biological replicates per condition.

Expression patterns for the 20 genes most differentially expressed in the conidiophore are presented in Fig. 11. Fourteen of these genes are consistent with those presented in Fig. 7, including the previously mentioned *ccg-2*, *ccg-1*, *ccg-15*, *acu-6*, and NCU04482. However, analyzing the conidiophore samples revealed 2 additional genes displaying striking differential expression patterns: NCU08769 (*con-6*) and NCU00265 (uncharacterized). The conidiation-specific gene *con-6* is also under clock control and is downregulated in Bulky conidiophores. NCU00265 encodes a predicted secreted protein and is upregulated in Bulky conidiophores.

## Discussion

Conidiation in *N. crassa* has been thoroughly investigated over the course of decades of study. While much work has been done to characterize the genetic, temporal, environmental, and circadian signals guiding conidiophore development, we lack a complete understanding of the morphological variation of these structures (Sargent and Kaltenborn 1972; Springer and Yanofsky 1989; Loros and Dunlap 2001). Recent work has begun to explore natural conidiophore variation, using a collection of wild isolates to identify 3 architectural phenotypes: WT, Wrap, and Bulky (Krach *et al.* 2020). We continued this exploration by investigating the impact of these phenotypes on sporulation and germination, developing a more robust model to estimate heritability of the trait, and identifying genes differentially expressed in representative strains for each conidiophore shape.

Previous work has demonstrated that conidiophore morphology impacts the distribution of distances traveled by sporulating conidia, or the “spore shadow” (Krach *et al.* 2020). Similar studies have revealed that dispersal patterns impact gene flow between natural populations of *Drosophila* and *Laelia rubescens*, for example Powell and Dobzhansky (1976) and Trapnell and Hamrick (2005). Modeled at a small scale, WT conidia displayed the lowest germination rate and traveled the least maximum distance following their sporulation (Krach *et al.* 2020). Here, we showed that both patterns were upheld at a larger scale, indicating that WT populations may not have as wide a colonization capacity, and thus as large of gene flow, as Wrap and Bulky (Fig. 1, Table 4). Other features of the spore, such as shape and a natural O-ring, affect spore dispersal (Roper *et al.* 2010; Fritz *et al.* 2013). Additional studies could be conducted to explore spore shadows from each conidiophore phenotype in different environments (Roper *et al.* 2010), such as on various plant substrates or in different climates.

We also demonstrated that conidiophore phenotype impacted germination rate and germination time of conidia on different carbon sources (Figs. 2 and 3). Germination of conidia from WT conidiophores drastically increased by 52% when fructose and glucose concentration decreased from 1% to 0.1%, presumably a stress response that was not observed in the other 2 phenotypes. On medium containing mannose or xylose as a carbon source, conidia from Bulky conidiophores showed a striking response, consistently germinating earlier and at significantly higher rates than the other 2 phenotypes. This suggests that Bulky is much more responsive than the other 2 strains to the stress of these unfavorable carbon sources.

Implementing tools previously developed in Krach *et al.* (2020), we used homokaryotic strains to more accurately estimate heritability of conidiophore phenotype. Our model suggested that at least 3 genes contribute to this complex trait with 2 epistatic interactions (Table 6). The fitted model revealed an estimated heritability of 0.47, an increase from the heritability previously estimated using the original, possibly heterokaryotic, wild isolates. To further explore the genes contributing to conidiophore morphology, we conducted RNA-seq on mycelia and conidiophores from each phenotype. The RNA-seq analysis is suggesting more complex genotype–phenotype interactions than the 3 interactions detected in Table 6.

Our RNA-seq results demonstrated that the Bulky (FGSC2229) transcriptional profile is unique from that of WT and Wrap, clearly separating it from the other 2 strains by PCA (Fig. 6). Five of the 20 most differentially expressed genes (Fig. 7) were *ccgs*, 3 of which showed distinctive expression patterns in the Bulky cell types: *ccg-2*, *ccg-1* (Lindgren 1994), and *ccg-14*. Notably, both *ccg-2* and *ccg-14* encode proteins that localize to the conidium cell wall. The CCG-2 protein, a hydrophobin, maintains hydrophobicity of the conidium cell wall (Bell-Pedersen *et al.* 1992). The protein encoded by *ccg-14* (*snodprot1*) bears sequence similarity to cerato-platanin, a phytotoxin prevalent in other ascomycetes that has both hydrophobin and expansin properties (Jeong *et al.* 2007). It is known that the cell wall plays a critical role in fungal morphology, growth rate, and viability, and several mutations affecting *N. crassa* cell wall components have been associated with a compact growth phenotype (Patel and Free 2019). Our study shows that compared to both WT and Wrap, Bulky mycelia and conidiophores have increased expression of *ccg-14* and decreased expression of *ccg-2*. It is possible that the Bulky cell wall is affected by the interaction of the CCG-2 and CCG-14 proteins, potentially affecting polarity of the structure and conidiophore morphology. An alternative hypothesis is that the Bulky strain has upregulated expression of the cerato-platanin phytotoxin as an evolved defense mechanism, another potential explanation for the compactness of the Bulky phenotype. Additional research should be conducted on known cell wall mutants and the role of CCG-14 in *Neurospora* to better assess the impact of these genes on morphology of the conidiophore.

Two genes with roles in metabolism were also differentially expressed in the Bulky strain: *acu-6* and NCU04482 (Fig. 7). *acu-6* is the structural gene for PEPCK, which converts oxaloacetate to phosphoenolpyruvate in gluconeogenesis (Flavell and Fincham 1968). Previous work has demonstrated that *acu-6* is upregulated under starvation and is responsive to quinic acid, an unfavorable carbon source (Tang *et al.* 2011). NCU04482 encodes a hypothetical protein and is upregulated by amino acid starvation (Schmoll *et al.* 2012). We observed that both loci are strikingly downregulated in Bulky mycelia and upregulated in Bulky conidiophores compared to corresponding cell types of the other 2 strains and constitute another interaction pair to consider. This provides evidence that the Bulky strain is more metabolically responsive to unfavorable carbon sources, such as quinic acid. This hypothesis is upheld by our germination assay results, where conidia from Bulky conidiophores had higher germination rates and earlier germination times on medium containing mannose or xylose as a carbon source (Figs. 2 and 3).

Comparisons between strain pairs revealed 5 loci consistently upregulated in Bulky, 4 of which are involved in communication: *doc-1*, *doc-2*, *plp-1*, and *plp-2* (Fig. 8). The *doc* genes identify CG compatibility prior to hyphal fusion, and the *plp* genes trigger germling-regulated death following an incompatible fusion (Heller *et al.* 2016, 2018). We did not find a correlation between CG assignment and conidiophore phenotype (Fig. 9). It is reasonable to suppose that there is more genic interactions taking place between these 4 genes in the Bulky conidiophore, where there is a higher density of cells than in WT or Wrap. As an example, Conidial anastomosis Tubes in *N. crassa* display density-dependent behavior (Roca *et al.* 2005). Enrichment of these *doc* and *plp* genes could also simply be a result of this increased communication due to density effects.

Alternatively, the Bulky strain may be more heterokaryotic than the WT or Wrap strain, requiring more communication and germling-regulated death following heterokaryon incompatibility. This hypothesis is supported by the fact that this Bulky strain has lower penetrance of the phenotype (46.55%) than the WT (77.78%) and Wrap (53.85%) strains selected for RNA-seq (Krach *et al.*, 2020). To test this hypothesis, RNA-seq could be conducted on homokaryotic F1s representing each conidiophore phenotype.

Identifying genes differentially expressed in just the conidiophore samples revealed many of the same loci differentially expressed according to both cell type and strain (Figs. 7 and 11). Among the 14 shared loci are *ccg-2*, *ccg-1*, *ccg-15*, *acu-6*, and NCU04482. This provides evidence that these genes were not solely identified by differences between cell types, but showed significant differential expression in the conidiophores and may play a role in the morphological differences observed. Analyzing differential expression patterns in the conidiophore samples did reveal 2 additional loci: *con-6* and NCU00265 (Fig. 11). Bulky conidiophores showed much lower expression of *con-6* and higher expression of NCU00265 than WT and Wrap conidiophores. While *con-6* has been well characterized as a light-responsive, clock-controlled, conidiation-specific gene, Δ*con-6* shows no obvious phenotype and the function of the CON-6 protein remains unknown (White and Yanofsky 1993; Olmedo *et al.* 2010). Additional work should be conducted to characterize the function of this protein and explore the possible connection of *con-6* downregulation and a Bulky phenotype. Further research should also be conducted on NCU00265 to better identify and characterize the predicted secreted protein it encodes. This protein has been detected alongside known cell wall proteins following their secretion by hyphae into growth medium (Maddi and Free 2010). If NCU00265 does encode a cell wall protein that is enriched in Bulky conidiophores, it provides additional evidence, alongside *ccg-2* and *ccg-14*, that the Bulky phenotype may in part be due to an interaction in its cell wall components.

Looking deeper into the differentially expressed transcripts revealed a more complex network of gene interactions. Gene set enrichment analysis of the top 500 differentially expressed genes revealed a significant upregulation of biological pathways known to be regulated by the clock (Supplementary Fig. 3A). A substantial proportion of ribosomal biogenesis is upregulated in considering the 2-factor differential expression model (Supplementary Fig. 3B). Clock outputs include genes with products heavily involved in multiple key cell processes including DNA metabolism, ribosome biogenesis in RNA metabolism, cell cycle, and protein metabolism (Dong *et al.* 2008; Al-Omari *et al.* 2018). While our most significantly differentially expressed genes were highlighted above, the enrichment analysis has revealed a deeper and more complex network of interactions with highly overlapping gene sets forming functional modules in the WT and Wrap phenotype compared to the Bulky phenotype.

High-throughput phenotyping has been recently applied in other fungal systems, such as *Saccharomyces cerevisiae*, to attribute genes to novel morphological phenotypes (Ohya *et al.* 2005). This study illustrates how high-throughput methods of phenotyping complex traits using machine learning applied to natural populations can be successfully combined with omics approaches, such as RNA-seq, to implicate the genes and their interactions underlying complex traits. The same approach is now being applied to characterize the morphology of arbuscular mycorrhizal fungi (AMF) in roots of sorghum in field populations to improve the production of biofuel because sorghum accessions grow differently in the presence of AMF (Watts-Williams *et al*., 2019). High-throughput phenotyping studies not only provide insights into the genetic basis of complex traits in natural and cultivated populations, but they also provide potential insights into the robustness of genetic systems to both environmental and genetic perturbations (Levy and Siegal 2008).

## Data availability

Transcriptome data are available at NCBI SRA Accession SRP353648. Code used to analyze the RNA-seq data with the RNA-seq data is publicly available on GitHub at https://github.com/michaelSkaro/Neurospora_crassa_transcriptomics.

Supplemental material is available at *G3* online.

## Supplementary Material

Jkac050_Supplementary_Figures

jkac050_Supplementary_Table_1
