# Supplementary material for: Characterizing the gene–environment interaction underlying natural morphological variation in Neurospora crassa conidiophores using high-throughput phenomics and transcriptomics
Source: G3 (Bethesda). 2022 Feb 28;12(4):jkac050. doi: 10.1093/g3journal/jkac050 (PMC8982394; doi:10.1093/g3journal/jkac050)
Supplement: Jkac050_Supplementary_Figures [file jkac050_supplementary_figures.docx]

SUPPLEMENTARY FIGURES


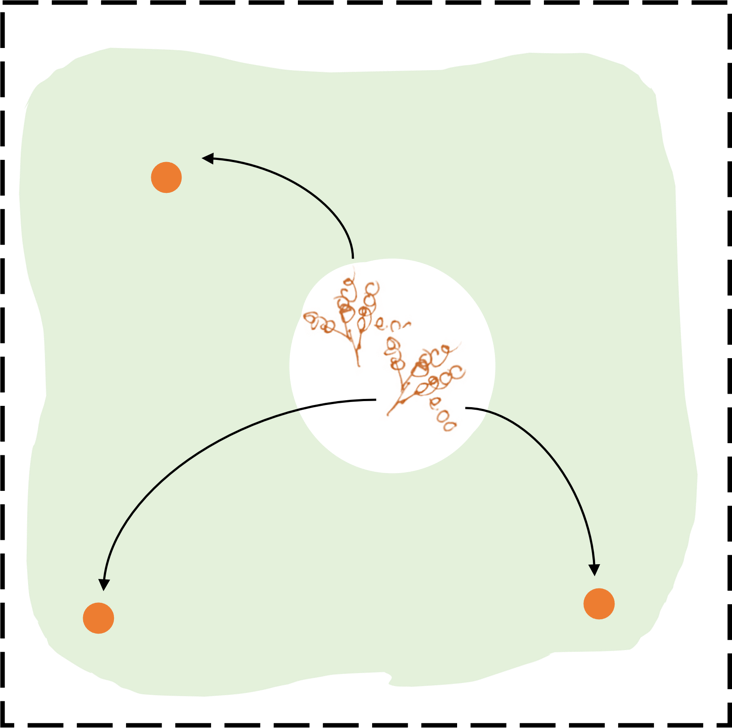


Supplementary Figure 1. Schematic of Sporulation Experiment. Mature conidiophores on a nitrocellulose membrane are placed at the center of a cake platter, which is surrounded by SFG medium. Conidia germinate and maintain colonial growth following sporulation from the center of the plate (Materials and Methods).


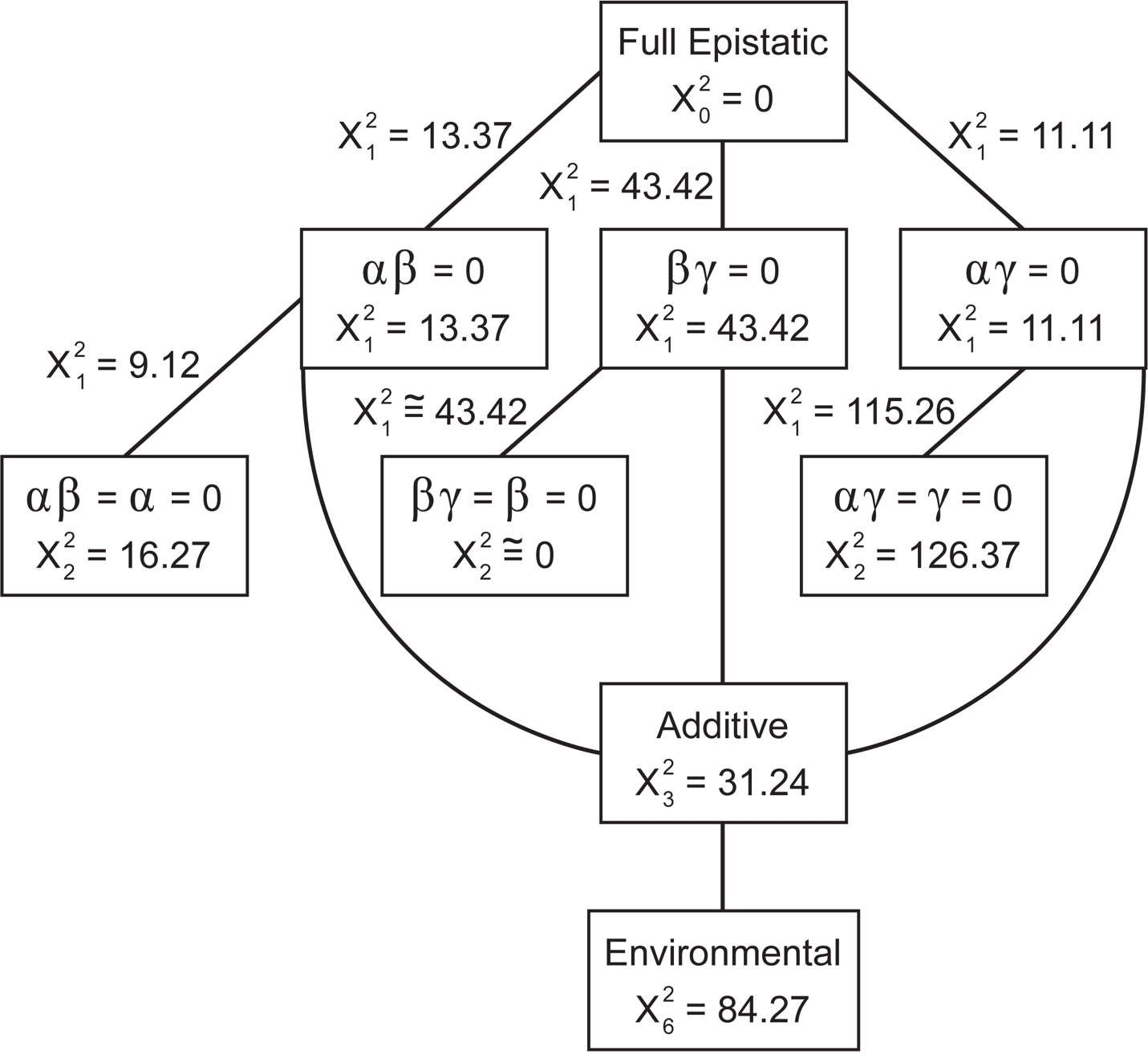


Supplementary Figure 2. A hierarchy of inheritance models is tested in which the simplest model has no genetic effects and the most complex model has epistatic pairwise interactions between all three loci. A chi-squared goodness of fit (X^2^) statistic is calculated for each model. The difference in chi-squared goodness of fit between a simpler model ($X_{H0}^{2})$ and a more complex model ($X_{HA}^{2})$ yields a chi-squared test of the simpler model (H0) vs the more complex model (HA). Chi-squared statistics within a box are for goodness fit. A chi-squared statistic on an edge between two models tests the simpler model below the edge with the more complex model above.

Supplementary Figure 3. Gene set enrichment analysis on the significantly differentially expressed genes. A. Up regulated processes known to be regulated by clock genes are significantly activated using a two-factor approach estimating the phenotypes effect on cell type in the design formula. The FGSC2229 for Bulky was used as the reference in the design matrix. B. Distribution of significantly enriched pathways in differentially expressed gene sets show substantial proportion of genes in pathways regulated by clock controlled genes upregulated. C. Enrichment map organized into a network with edges connecting overlapping gene sets. Mutually overlapping gene sets clustered together identify functional modules activated in WT and Wrap phenotypes.

|  | Accuracy | Precision | Recall |
| --- | --- | --- | --- |
| Train | 0.9139 | 0.9158 | 0.9139 |
| Validation | 0.7667 | 0.7722 | 0.7667 |
| Test | 0.6667 | 0.6950 | 0.6667 |

Supplementary Table 1. Evaluation of the fine-tuned model. The classification model was fine-tuned with new samples. Accuracy, precision, and recall of different separation groups are presented. Evaluation is defined in Krach et al. 2020.
