# Supplementary material for: Characterizing the gene–environment interaction underlying natural morphological variation in Neurospora crassa conidiophores using high-throughput phenomics and transcriptomics
Source: G3 (Bethesda). 2022 Feb 28;12(4):jkac050. doi: 10.1093/g3journal/jkac050 (PMC8982394; doi:10.1093/g3journal/jkac050)
Supplement: jkac050_Supplementary_Table_1 [file jkac050_supplementary_table_1.docx]

Supplementary Table 1. Evaluation of the fine-tuned model. The classification model was fine-tuned with new samples. Accuracy, precision, and recall of different separation groups are presented. Evaluation is defined in Krach et al. 2020.

|  | Accuracy | Precision | Recall |
| --- | --- | --- | --- |
| Train | 0.9139 | 0.9158 | 0.9139 |
| Validation | 0.7667 | 0.7722 | 0.7667 |
| Test | 0.6667 | 0.6950 | 0.6667 |
